# Evaluation of the effects of anthelmintic administration on the fecal microbiome of healthy dogs with and without subclinical *Giardia* spp. and *Cryptosporidium canis* infections

**DOI:** 10.1371/journal.pone.0228145

**Published:** 2020-02-06

**Authors:** Madeline A. Fujishiro, Jonathan A. Lidbury, Rachel Pilla, Jörg M. Steiner, Michael R. Lappin, Jan S. Suchodolski

**Affiliations:** 1 Small Animal Clinical Sciences, Gastrointestinal Laboratory, College of Veterinary Medicine and Biomedical Sciences, Texas A&M University, College Station, Texas, United States of America; 2 Center for Companion Animal Studies, Colorado State University, Fort Collins, Colorado, United States of America; University of Illinois, UNITED STATES

## Abstract

**Background:**

The gastrointestinal microbiome plays an important role in host health and there is increasing concern regarding the deleterious effects of pharmaceuticals on the fecal microbiome. The effect of anthelmintic therapy on the fecal microbiome in dogs has not yet been evaluated. The purpose of this study was to evaluate the effect of anthelmintic administration on the fecal microbiome of dogs with and without subclinical *Giardia* species and *Cryptosporidium canis* infections.

**Methodology/Principal findings:**

Part 1: 6 healthy adult research beagles with subclinical giardiasis and cryptosporidiosis were administered a commercially available preparation of febantel combined with pyrantel and praziquantel (FPP) orally daily for three days. Part 2: 19 healthy staff-owned dogs without giardiasis or cryptosporidiosis were divided into a treatment group (n = 9) that was administered fenbendazole orally daily for five days and an untreated control group (n = 10). For both parts of the study, feces were collected at multiple time points before and after anthelmintic (FPP or fenbendazole) administration. Fecal DNA was extracted for Illumina sequencing of the bacterial 16S rRNA gene and qPCR assays. Neither FPP nor fenbendazole treatment caused a significant change in alpha or beta diversity or the relative abundance of bacterial species. Upon univariate statistical analysis neither FPP or fenbendazole caused minimal changes in the fecal microbiota.

**Conclusion:**

FPP administration was associated with minimal alterations of the fecal microbiome of healthy research beagles with subclinical giardiasis and cryptosporidiosis. Fenbendazole administration was associated with minimal alterations of the fecal microbiome of healthy staff owned dogs.

## Introduction

The gastrointestinal (GI) microbiome is a complex ecosystem that plays an important role in host health and immunity. It stimulates the host’s immune system, defends against enteropathogens, and offers nutritional benefits [1]. It is affected by multiple factors, including dietary influences, gastrointestinal secretions and motility, mucosal barrier integrity, lymphatic tissue, and bacterial interactions [2]. The microbiome is dynamic and subject to change due to diverse mechanisms, including disease states and medical therapies. Intestinal dysbiosis in dogs has been associated with a number of disorders including acute and chronic enteropathies, exocrine pancreatic insufficiency, and intestinal parasitism [3–6]. Additionally, concerns regarding the effects of pharmaceuticals on the bacterial microbiota in humans and veterinary species have been raised, more specifically concerning the potential deleterious effects of antimicrobials [7]. In humans, negative health events in childhood (e.g. antibiotic use, malnutrition, premature birth) can lead to abnormal development of the intestinal microbiome and disruptions of the GI microbiome have been associated with multiple potential consequences, including inflammatory bowel disease, obesity, type II diabetes, and celiac disease [8, 9].

*Giardia* species (spp.) and *Cryptosporidium canis* infections are common in dogs throughout the world and can occur as single infections or coinfections [10, 11]. The most common clinical sign observed with either infection is small bowel diarrhea, but this is usually associated with young animals or those in crowded environments as most dogs are colonized with no signs of disease [10]. The experience of the authors is that *C*. *canis* is not very pathogenic and that for dogs co-infected with *Giardia* spp., treatment of the *Giardia* spp. infection alone is usually adequate for controlling clinical signs of disease. A previous study evaluating subclinical *Giardia*-positive and *Giardia*-negative dogs found a significant difference in bacterial genera between groups; however, the effect of *Giardia* spp. infection with *C*. *canis* co-infection or the treatment of these infections with an anthelminthic agent on the fecal microbiome of dogs has not yet been characterized [6].

Metronidazole is commonly used to treat giardiasis in dogs and cats [12, 13]. Recently, a study evaluating the fecal microbiome of healthy dogs showed that short-term use of this antibiotic can cause a dysbiosis [14]. In addition, metronidazole can cause neurological signs in dogs and cats [15, 16]. Fenbendazole and febantel are broad spectrum benzimidazole anthelmintics that have been used as alternative drugs for the treatment of giardiasis [17, 18]. There is a commercially available preparation of febantel that is combined with pyrantel and praziquantel (FPP; Drontal^®^Plus; Bayer Animal Health, Shawnee, KS) that is labeled for the treatment of *Giardia* spp. infections in dogs in some countries [19]. To the authors’ knowledge, the effect of administration of an anthelmintic agent (combination product FPP or fenbendazole) on the fecal microbiome of dogs has not yet been described. However, fenbendazole has not been shown to have a major effect on the hindgut microbiota of horses [20] or the gut microbiome of mice [21] and a moxidectin praziquantel combination product did not have a major effect on the fecal microbiota of horses [22].

This study was performed in two parts. The objectives of part 1 of the study were to evaluate the fecal microbiome in research beagles with chronic, subclinical *Giardia* spp. and *C*. *canis* infections and to determine if there were alterations in the fecal microbiome after administration of FPP. The objective of part 2 of the study was to further investigate the effect of fenbendazole on the fecal microbiome of healthy client-owned dogs. As to our knowledge fenbendazole, febantel, pyrantel, or praziquantel have not been shown to affect the gastrointestinal microbiome in other species, we hypothesized that substantial changes in the fecal microbiome after administration of either FPP or fenbendazole would not be identified in dogs.

## Materials and methods

### Ethics statement

Part 1 of the study was approved by the Institutional Animal Care and Use (IACUC) committee of the contract research facility based in Fort Collins, Colorado where this work was performed (AUP: 170.031). Part 2 of the study was reviewed and approved by the IACUC at Texas A&M University (AUP: 2017–0186 CA). Written informed client consent was acquired prior to enrollment of these dogs.

### Animals and sample collection

#### Part 1: Effect of febantel combined with pyrantel and praziquantel on the fecal microbiota of research beagles

*Animals*. Six young (3 male; 3 female) healthy purpose-bred beagles with subclinical *Giardia* spp. and *C*. *canis* infections and a normal complete blood cell count (CBC) and serum biochemical profile were included in the study. The dogs were housed individually in pens that were approximately 36 square feet in size for the duration of the study. The dogs were fed the same standardized facility kibble throughout the study and were exercised and socialized twice daily. Hard rubber toys were provided for additional enrichment. At the end of the study, the dogs were adopted to private homes or returned to the colony at the contract research facility.

*Clinicopathological analysis*. Prior to enrollment, blood (6 mL) was collected by jugular venipuncture using minimal manual restraint. After venipuncture, the blood was placed into EDTA or allowed to clot prior to serum separation for performance of a CBC and serum biochemical profile (Veterinary Diagnostic Laboratory, Colorado State University, Fort Collins, Colorado).

*Anthelmintic administration*. The commercially available preparation of febantel, pyrantel, and praziquantel (Drontal^®^Plus; Bayer Animal Health, Shawnee, KS) was administered as directed by the manufacturer by mouth, daily, for 3 days with approximately 1 tablespoon of canned dog food to promote ingestion (days 0, 1 and 2 of the study). This resulted in the administration of febantel at the range of 27.8–34.2 mg/kg per dose, pyrantel at the range of 5.6–6.9 mg/kg per dose, and praziquantel at the range of 5.6–6.9 mg/kg per dose.

**Fig 1:**
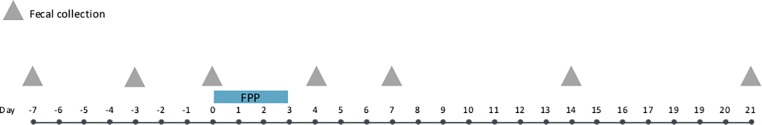
Study timeline of six healthy adult research beagles with subclinical giardiasis and cryptosporidiosis. Dogs were administered a commercially available preparation of febantel combined with pyrantel and praziquantel (FPP) orally on days 0, 1 and 2. At each fecal collection, fluorescent antibody staining for *Giardia* cysts and *Cryptosporidium* oocysts, fecal flotation (with the exception of day-7), and DNA extraction (with the exception of day 7) were performed.

*Collection of fecal samples*. All fecal samples collected for analyses were passed by natural defecation into the pens. Feces were collected from the pens within 8 hours of being passed on study days -7, -3, 0, 4, 7, 14, and 21 (Fig 1). This delay between elimination of the feces and processing is not expected to have affected the results as it was previously shown that the storage of cat feces for up to 3 days at ambient temperatures did not affect microbiome structure or membership [23]. A fecal score was determined by 1 of 2 trained observers using a previously standardized scoring system (Purina^®^ Fecal Scoring System, Nestle Purina PetCare Co., St. Louis, MO, USA). Fluorescent antibody staining for *Giardia* cysts and *Cryptosporidium* oocysts (Giardia/Cryptosporidium immunofluorescence assay, MERIFLUOR^®,^ Meridian Diagnostics, Akron, Ohio) were performed on each sample from days -7, -3, 0, 4, 7, 14, and 21. In addition, microscopic examination of feces after sugar centrifugation was performed on each fecal sample except those collected on day -7. While being processed for parasite examination, an aliquot of feces from days -7, -3, 0, 4, 14, and 21 was stored at -80°C until shipment to the Gastrointestinal Laboratory at Texas A & M University on dry ice for DNA extraction.

#### Part 2: Effect of fenbendazole on the fecal microbiota of healthy staff owned dogs

*Animals*. Healthy adult dogs belonging to faculty and staff at Texas A&M University were recruited. Dogs of any size, sex, or breed that were aged greater than 9 months and less than 10 years of age were eligible for inclusion. Blood (8 mL) was collected prior to enrollment by jugular or saphenous venipuncture using minimal manual restraint. Urine (5 mL) was first attempted to be collected by free catch and then obtained by cystocentesis if the dog would not voluntary void. The blood was placed into EDTA or allowed to clot prior to serum separation and the urine was placed in a sterile tube. A CBC, serum biochemical panel, and urinalysis were performed at the Texas Veterinary Medical Diagnostic Laboratory, College Station, Texas. Spontaneously passed feces were collected by the dog’s owner. The health of the dogs was determined using an owner questionnaire (S1 Questionnaire), physical examination, and results from a complete blood count, serum biochemical panel, urinalysis, and fecal flotation as well as fluorescent antibody staining for *Giardia* cysts and *Cryptosporidium* oocysts as previously described. Dogs were excluded if they had a history of gastrointestinal disease (i.e., vomiting, regurgitation, diarrhea, or anorexia) lasting greater than two consecutive days in the preceding three months, antimicrobial treatment in the previous six months, systemic non-gastrointestinal disease (e.g., chronic kidney disease, congestive heart failure, etc.), received medications other than routine prophylactics (heartworm, flea, or tick prevention), or tested positive for intestinal parasites on days -7 and -1 of the study. Twenty-one dogs were initially evaluated for inclusion, but two dogs were excluded because of intestinal parasitism (one dog tested positive for *Giardia* spp., another dog tested positive for both *Giardia* spp. and *Ancylostoma caninum*). The remaining nineteen dogs were randomly assigned to the treatment group (n = 9) or the control group (n = 10). Dogs were fed their current commercial maintenance diets for the duration of the study.

*Anthelmintic administration*. Dogs in the treatment group were administered fenbendazole (50 mg/kg) by mouth, once daily for five consecutive days starting on day 0. The dogs in the control group did not receive any treatment.

**Fig 2:**
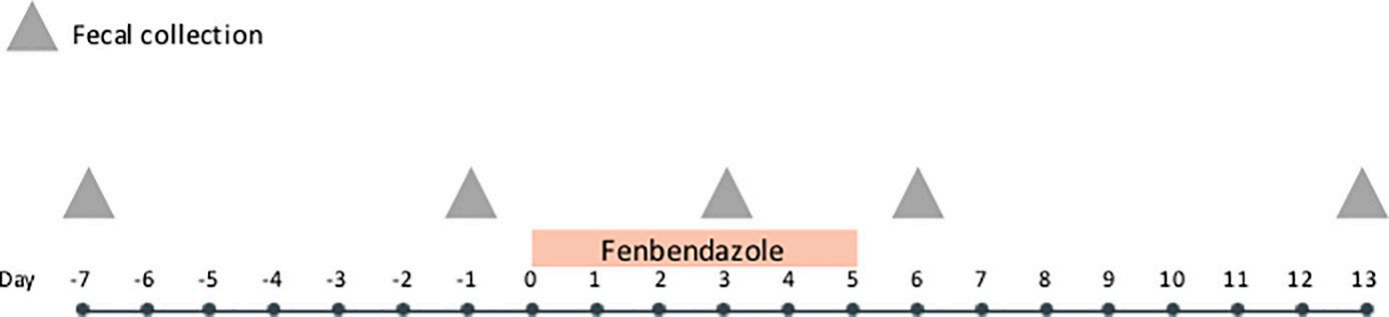
Study timeline of 19 healthy staff-owned dogs treated with fenbendazole (n = 9) and untreated control group (n = 10). Fenbendazole (50 mg/kg) was administered to dogs in the treatment group on days 0–4. Fluorescent antibody staining for *Giardia* cysts and *Cryptosporidium* oocysts and fecal flotations were performed on days -7 and -1. DNA was extracted from an aliquot of feces from days -7, -1, 3, 6, and 13.

*Fecal collection*. All fecal samples collected for analyses were passed by spontaneous voiding at the owner’s property or on hospital premises. After collection the owners refrigerated the feces at 4°C and submitted the specimen to the laboratory within 12 hours. Feces were collected on study days -7, -1, 3, 6, and 13 (Fig 2). A fecal score was determined as previously described. Fluorescent antibody staining for *Giardia* cysts and *Cryptosporidium* oocysts was performed on each sample from days -7 and -1. Additionally, a microscopic examination of feces after sugar centrifugation was performed on an aliquot of feces from days -7 and -1 at a commercial laboratory (TVMDL, College Station, Texas). DNA was extracted from an aliquot of feces from days -7, -1, 3, 6, and 13.

### DNA extraction

DNA was extracted from the aliquots of feces saved from days -7, -3, 4, 14, and 21 in part 1 and days -7, -1, 3, 6, and 13 in part 2 of the study. DNA was extracted using the MoBio Power soil DNA isolation kit (MoBio Laboratories, USA) according to the manufacturer’s instructions.

### Quantitative PCR assays

Quantitative PCR (qPCR) assays were performed as previously described [3, 24, 25]. The qPCR data was expressed as the log amount of DNA (fg) for each particular bacterial group/10 ng of isolated DNA [24, 25]. The qPCR assays were used to assess abundance of total bacteria, *Faecalibacterium*, *Turicibacter*, *Escherichia coli*, *Streptococcus*, *Blautia*, *Fusobacterium*, and *Clostridium hiranonis* and to calculate a previously published dysbiosis index (DI) [25]. PCR assays for *Clostridioides difficile* (formerly, *Clostridium difficile*) *and Clostridium perfringens* were performed using the *C*. *difficile*–TaqMan assay and TaqMan *C*. *perfringens* enterotoxin gene as previously described [26–28].

### 16S rRNA sequencing

Illumina sequencing of the bacterial 16S rRNA genes was performed using primers 515F-Y (5’-GTGYCAGCMGCCGCGGTAA-3’) to 806RB (5’- GGACTACNVGGGTWTCTAAT-3’) at the MR DNA laboratory (www.mrdnalab.com, Shallowater, TX, USA).

### Analysis of 16S rRNA genes

Sequences were processed and analyzed using a Quantitative Insights Into Microbial Ecology 2 (QIIME 2) v 2018.6 pipeline. Briefly, raw sequence data was screened, trimmed, denoised with dada2, chimera depleted, and quality filtered. Operational taxonomic units (OTUs) were defined as sequences with at least 97% similarity with Greengenes v.13.8 database. Samples from part 1 and part 2 of the study were rarefied to 17,050 and 15,309 sequences per sample to account for unequal sequencing depth, respectively. The rarefaction depth was based on the lowest read depth of samples to have the optimum combination between number of sequences and number of samples in the diseased group. Alpha diversity was measured with the Chao1 (richness), Shannon diversity, and observed Operational Taxonomic Unit (OTU) metrics. Beta diversity was evaluated with the phylogeny based UniFrac distance metric and visualized using Principal Coordinate Analysis (PCoA) plots [29]. The raw sequences were uploaded to the NCBI Sequence Read Archive under the accession numbers SRP162534 and SRP163138 for part 1 and part 2 of the study, respectively.

### Statistical analysis

#### qPCR data

Data was assessed for normality using the Kolmogorov-Smirnov test and visual inspection of frequency distribution histograms. For part 1, log DNA for each of the measured bacterial groups and DI were compared across time points using the repeated measures ANOVA. Post-hoc testing was performed with Tukey’s test as appropriate. Fecal scores were compared across time points using Friedman’s test. For part 2, a mixed effects model was used to evaluate the effects of time point, treatment group, and their interaction on log DNA for each of the measured bacterial groups and DI. Post-hoc testing was performed as appropriate with Tukey’s test. Statistical significance was set as *p* < 0.05. Statistical analysis was performed using GraphPad Prism v7.0 (GraphPad Software, San Diego, California).

#### Sequencing data

Univariate analysis was performed at each taxonomic level with Friedman’s test and corrected for false discoveries. An adjusted p-value (q-value) < 0.05 was considered statistically significant. Post hoc Dunn's multiple comparison test was used to determine the time points in which bacterial taxa that were significantly altered by treatment. Analysis of Similarity test (ANOSIM) was performed with PRIMER 6 software package (PRIMER-E Ltd., Luton, UK) to analyze significant differences in microbial communities after treatment with FPP or fenbendazole in part 1 and part 2, respectively.

## Results

### Clinical and parasitology findings

#### Part 1

All dogs willingly ingested FPP and no adverse effects were noted at any time point. The fecal scores and parasitology test results of the six beagles in part 1 are available in the supplementary data (S1 Table). None of the dogs had diarrhea during the study; all fecal scores were <4 with the exception of one dog on day 0 (dog F), one dog on all post-treatment days (dog D), and one dog on day 14 (dog C). Fecal scores did not change over time (*p* = 0.7894). When all pretreatment results were combined, it was shown that all 6 dogs were co-infected with *C*. *canis*. Post-treatment, one dog was positive for *Giardia* spp. cysts and *C*. *canis* oocysts on Day 14 (dog A); the remaining five dogs were negative at each post-treatment time point.

#### Part 2

The characteristics of dogs in part 2 of the study are summarized in Table 1. All dogs in the treatment group (n = 9) were administered fenbendazole on days 0–4 with no clinical side-effects noted at the time of administration. All fecal scores over the course of the study were <4 with the exception of one control dog on day 13 that had a fecal score of 4. There was no significant effect of treatment group (*p* = 0.4438), time point (*p* = 0.3152), or their interaction (*p* = 0.6994) on fecal score.

**Table 1:** Characteristics of 19 healthy staff-owned dogs treated with fenbendazole (n = 9) and an untreated control group (n = 10).

Dog Characteristics	Control	Treatment
Number	10	9
Mean age ± SD (years)	6.7 ± 1.7	5.1 ± 2.6
Gender (male/female)	(7/3)	(5/4)
Breed	Mixed-breed (3) Pitbull Terrier (2) Standard Poodle (1) Australian Shepherd (1) Labrador Retriever (1) Miniature Dachshund (1) Italian Greyhound (1)	Mixed-breed (3) Border Collie (2) Rhodesian Ridgeback (1) Australian Shepherd (1) Shih Tzu (1) German Shepherd (1)
Diet	Purina ProPlan Chicken & Rice Dry (3), Purina ProPlan Lamb & Rice Dry (2), Eukanuba Adult Maintenance Dry (1), Purina One Chicken & Rice Dry, Purina O/M Dry (1), Purina Adult Dog Chow Chicken (1), Hill’s Science Diet Adult Maintenance Chicken (1), unknown (1)	Purina ProPlan Chicken & Rice Dry (5), unknown (1), Hill’s Prescription Diet j/d Dry, Purina Pro Plan Small Breed Dry and Hill’s Science Diet Canned—Chicken & Beef (1), Purina Adult Dog Chow Chicken Dry (1), Hill’s Prescription Diet i/d (1)

### qPCR assays

#### Part 1

**Fig 3:**
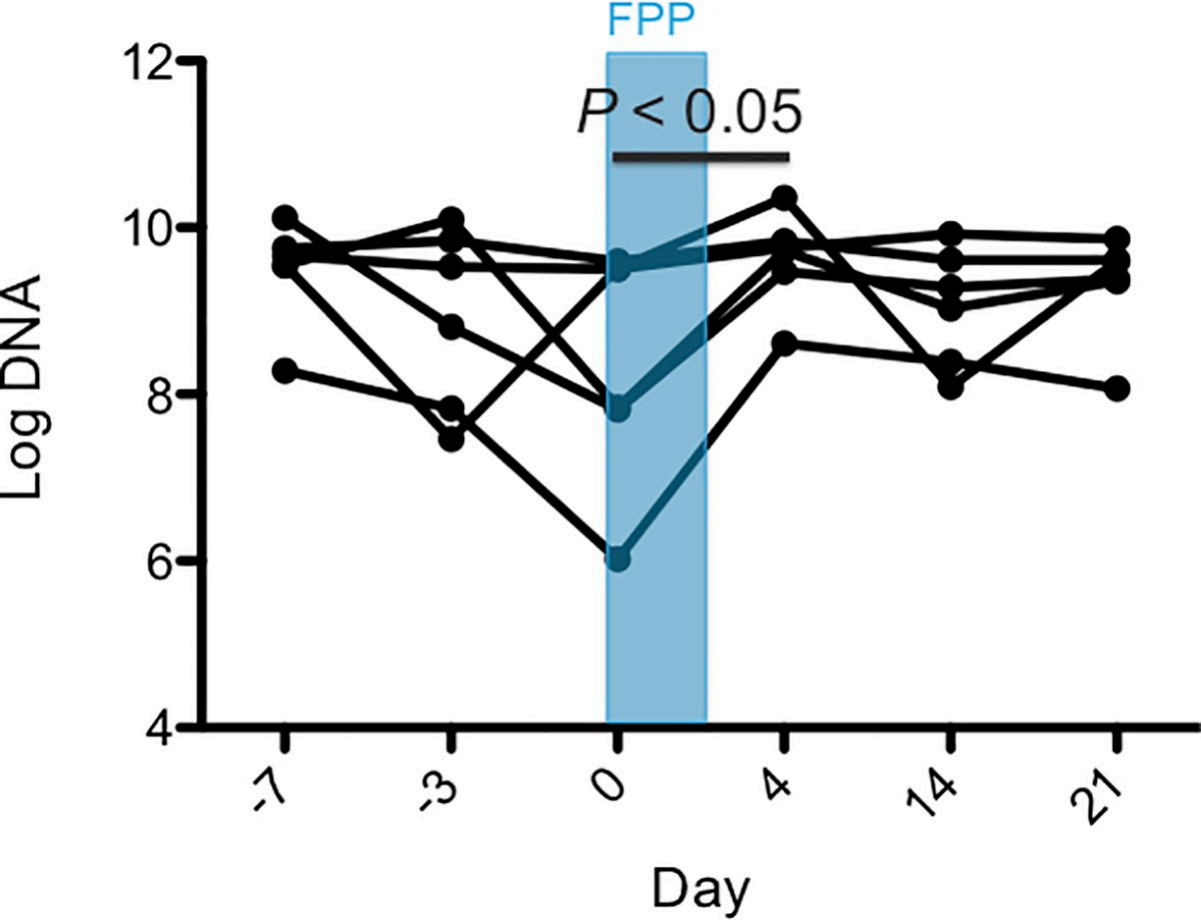
Fecal abundance of *Fusobacterium* in dogs receiving febantel combined with pyrantel and praziquantel (FPP). Days are represented on the X-axis and log DNA (the log amount of DNA (fg) for each particular bacterial group/10 ng of isolated DNA) on the Y-axis. Each dot represents an individual dog on each study day. Days -7 and -3 are pre-treatment, the treatment period with FPP is on days 0 to 2, and days 4, 14, and 21 are post-treatment. The abundance of *Fusobacterium* increased significantly between day 0 and day 4 (*p* < 0.05).

**Fig 4:**
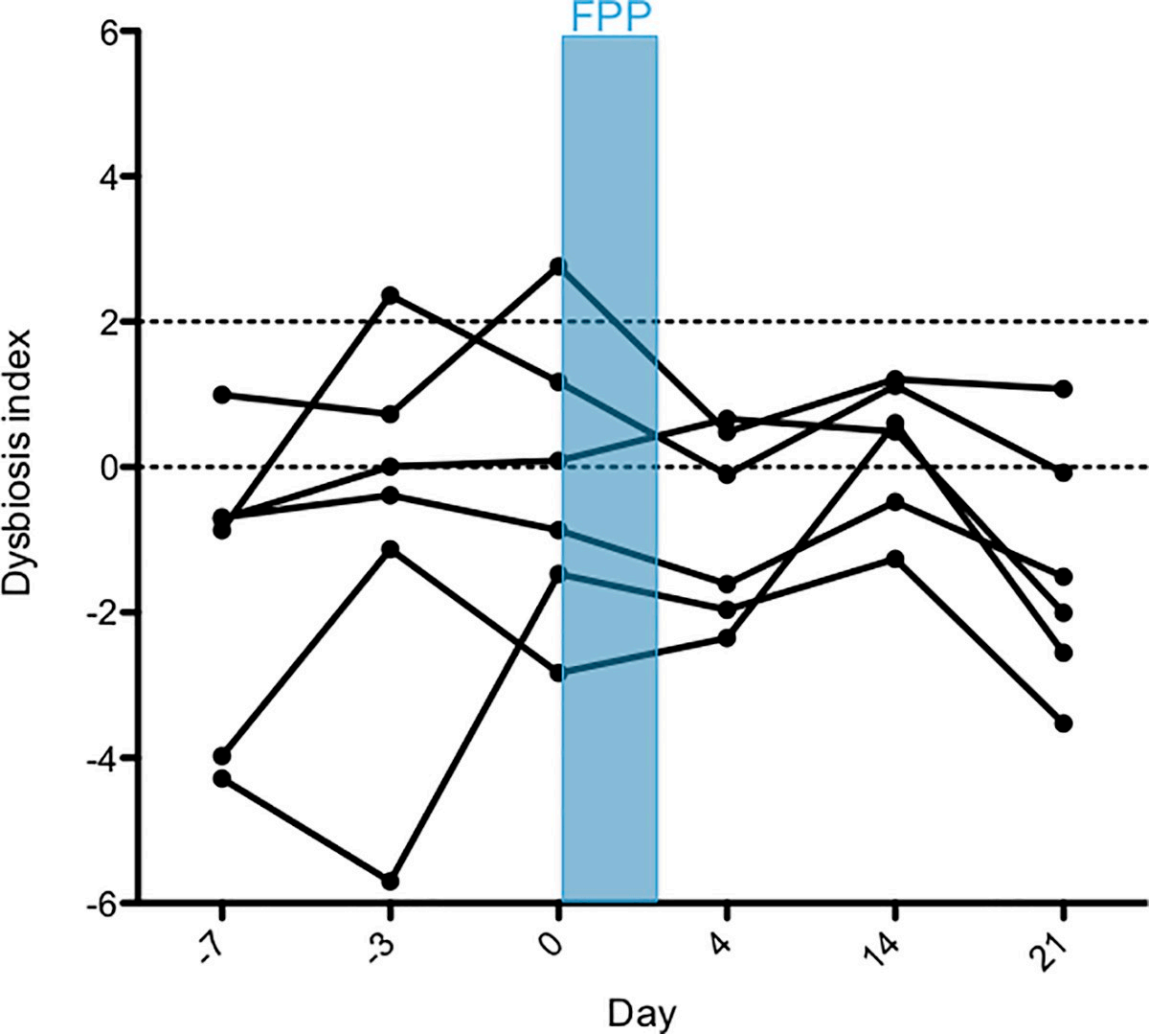
Fecal dysbiosis index of dogs receiving febantel combined with pyrantel and praziquantel (FPP). Days are represented on the X-axis and the mean (±SD) dysbiosis index on the Y-axis. The DI range between 0 and 2 is considered equivocal, and a DI greater than 2 suggests dysbiosis. Each dot represents an individual dog on each study day. Days -7 and -3 are pre-treatment, the treatment period with FPP is on days 0 to 2, and days 4, 14, and 21 are post-treatment. Overall, there was a significant difference of the DI across all time points (*p* = 0.0485) but on post-hoc testing there was no significant difference in DI between any of the time points.

No significant changes over time were found for the fecal abundances of bacteria using universal primers (*p* = 0.9109), *Fecalibacterium* (*p* = 0.0617), *Turicibacter* (*p* = 0.4405), *Streptococcus* (0.1474), *E*. *coli* (*p* = 0.4710), *Blautia* (*p* = 0.2526), or *C*. *hiranonis (p =* 1.083). An increase in the fecal abundance of *Fusobacterium* between days 0 and 4 (*p* < 0.05; Fig 3) was found. These qPCR assay results were used to calculate the DI (Fig 4). Overall there was significant differences of the DI across all time points (*p* = 0.0485) but on post-hoc testing there was no significant difference of the DI between any of the time points. There was no significant change in the fecal abundance of *C*. *perfringens* across time points (*p* = 0.1489) and no dog had a measurable fecal abundance of *C*. *difficile* at any time point.

#### Part 2

**Fig 5:**
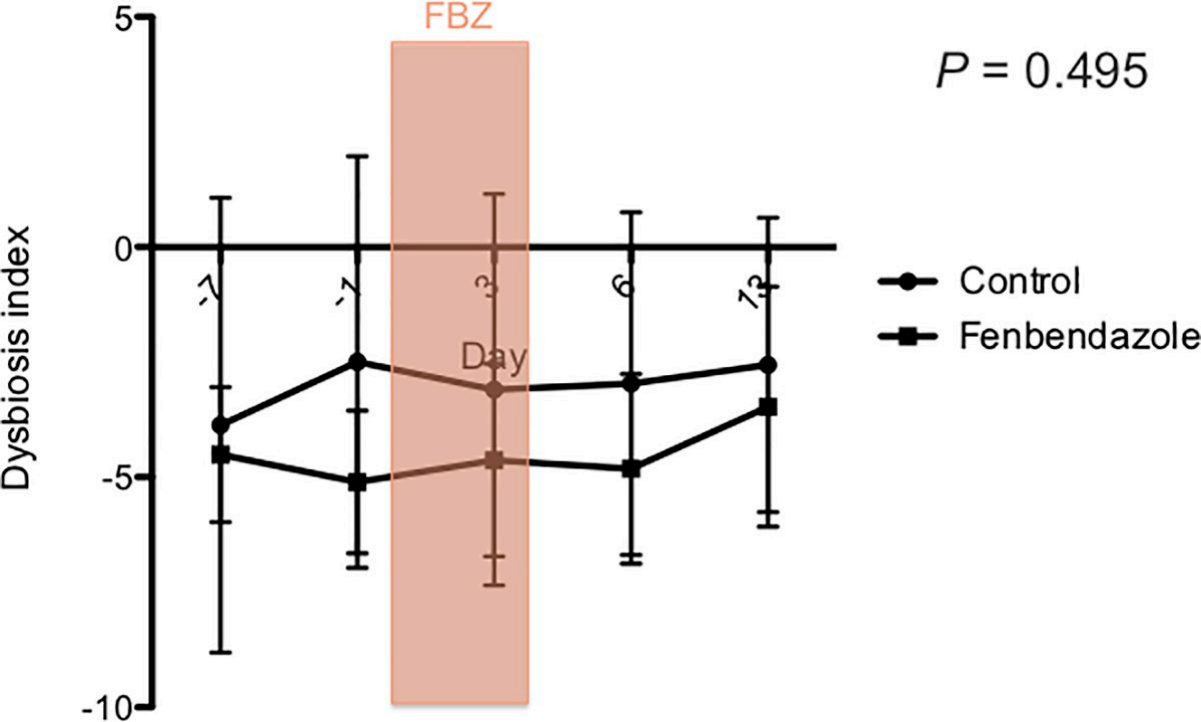
Fecal dysbiosis index in dogs after administration of oral fenbendazole and in untreated controls. Days are represented on the X-axis and the mean (±.SD) dysbiosis index on the Y-axis. The DI range between 0 and 2 is considered equivocal, and a DI greater than 2 suggests dysbiosis. Days -7 and -1 are pre-treatment, days 0 and 4 during treatment with fenbendazole (FBZ), and days 6 and 13 are post-treatment. No significant changes in the DI were observed between groups or across time points.

**Fig 6:**
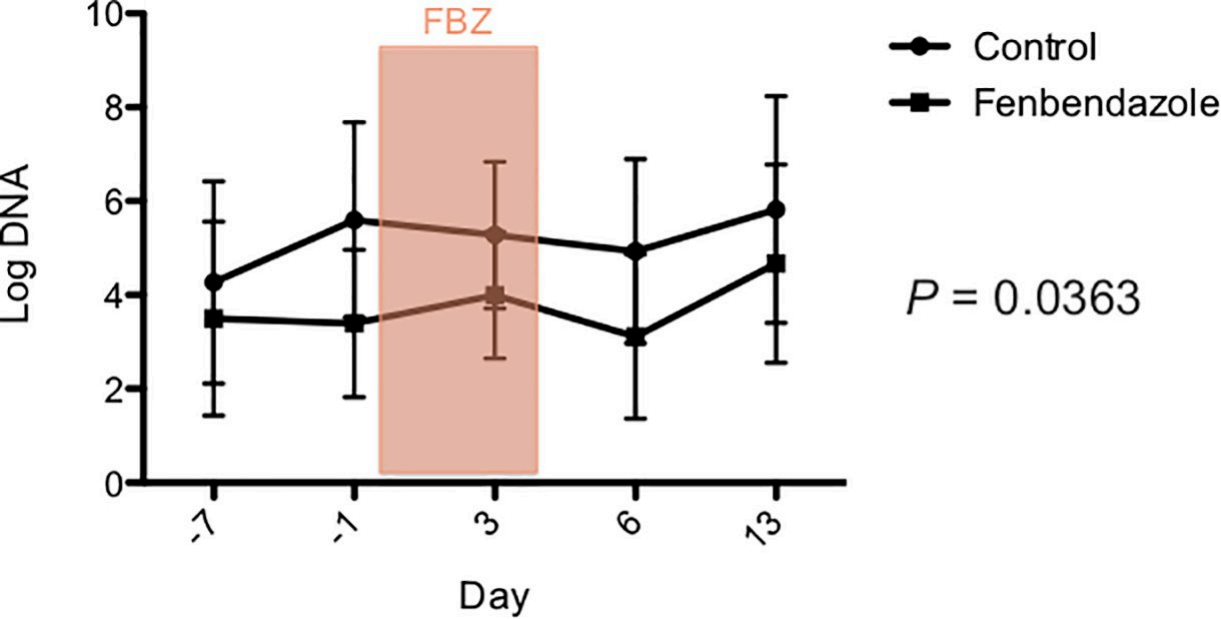
*E*. *coli* qPCR assay after administration of oral fenbendazole and in untreated controls. Days are shown on the X-axis and mean (± SD) log DNA (the log amount of DNA (fg) for each particular bacterial group/10 ng of isolated DNA) on the Y-axis. Days -7 and -1 are pre-treatment, days 0 and 4 during treatment with fenbendazole (FBZ), and days 6 and 13 are post-treatment. After correcting for multiple comparisons, there were no significant differences between groups for any of the time points.

**Fig 7:**
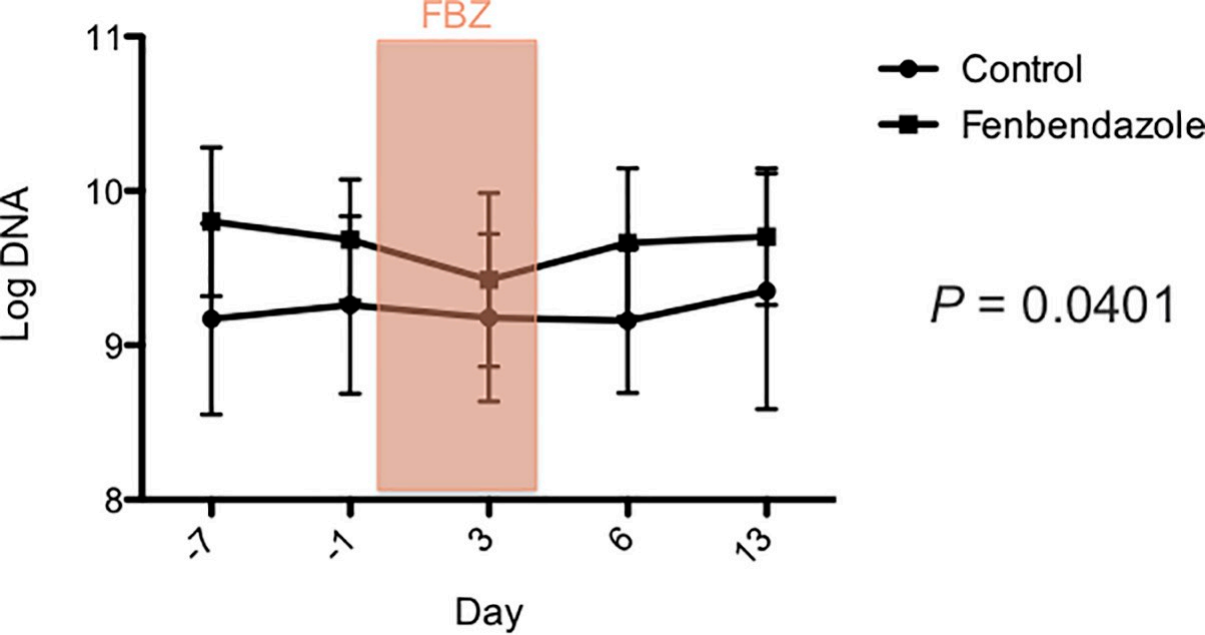
*Fusobacterium* qPCR assay after administration of oral fenbendazole and in untreated controls. Days are shown on the X-axis and mean (± SD) log DNA (the log amount of DNA (fg) for each particular bacterial group/10 ng of isolated DNA) on the Y-axis. Days -7 and -1 are pre-treatment, days 0 and 4 during treatment with fenbendazole (FBZ), and days 6 and 13 are post-treatment. After correcting for multiple comparisons, there was no significant difference between groups at any of the time points.

There was no significant effect of treatment group, day, or their interaction on the DI (Fig 5), and the fecal abundances, *Faecalibacterium*, *Turicibacter*, *Streptococcus*, *Blautia*, *C*. *hiranonis* and total bacteria DNA using universal primers (S2 Table). There was a significant effect of treatment group for *E*. *coli* (*p* = 0.0363; Fig 6) and *Fusobacterium* (*p* = 0.0401; Fig 7). However, after correcting for multiple comparisons, there were no significant differences between treatment groups at any time point for either bacterial group. Furthermore, there was no significant effect of day (*p* = 0.0585, *p* = 0.7927), or the interaction between treatment and day (*p* = 0.8236, *p* = 0.3624) for *E*.*coli* and *Fusobacterium*, respectively. One dog in the control group had a measurable fecal abundance of *C*. *difficile*, but the other dogs in each group did not. There was no significant effect of treatment group, day, or their interaction on the fecal abundance of *C*. *difficile* or *C*. *perfringens*.

### Sequence analysis

#### Part 1

The sequence analysis yielded 1,113,653 quality sequences for all analyzed samples (n = 36, mean ± SD = 31,490.361 ± 9,849.815) after removing chimeras and singletons. The samples were rarefied at 17,050 sequences per sample for even-depth analysis.

#### Part 2

The sequence analysis yielded 3,264,068 quality sequences for all analyzed samples (n = 100, mean ± SD = 32,640.680 ± 7,405.940) after removing chimeras and singletons. The samples were rarefied at 15,309 sequences per sample for even-depth analysis.

### Alpha diversity

#### Part 1

Alpha diversity, which assesses diversity within a sample, was described by species richness, Chao 1, and Shannon diversity index. No significant differences were found across time points.

#### Part 2

Alpha diversity, described by species richness, Chao 1, and Shannon diversity index, did not find any significant differences between groups and/or time points.

### Beta diversity

#### Part 1

**Fig 8:**
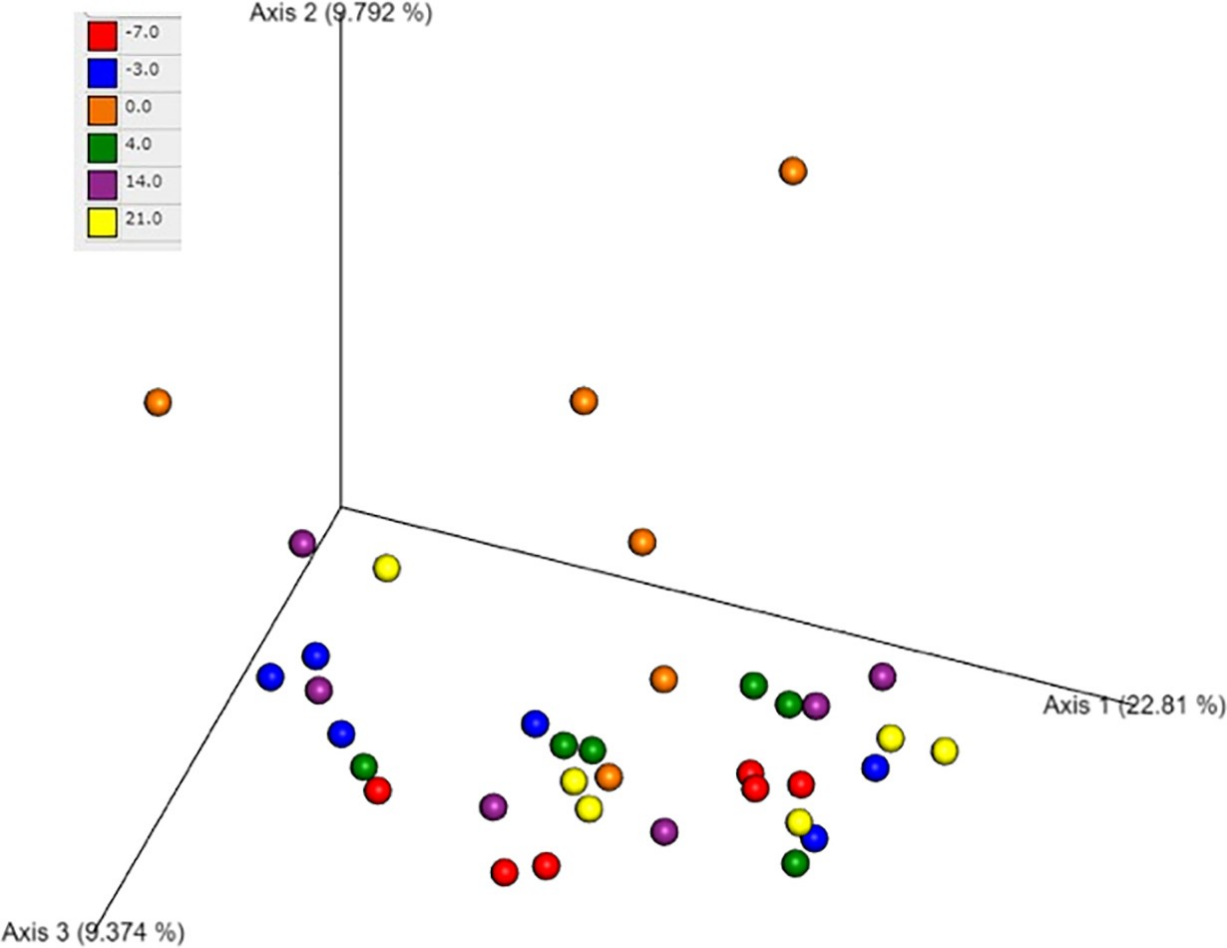
Principal coordinates analysis (PCoA) of microbial communities from the fecal samples of dogs after administration of oral febantel combined with pyrantel and praziquantel. The figure shows a 3D PCoA plot based on unweighted UniFrac distances of 16S rRNA genes. Each of the days is represented by a different color. There is no clustering of dogs by time point.

Beta diversity compares the bacterial communities between each sample and was assessed by principal coordinates analysis (PCoA) plots. There was no significant difference of microbial communities across time points based on an ANOSIM (Fig 8).

#### Part 2

**Fig 9:**
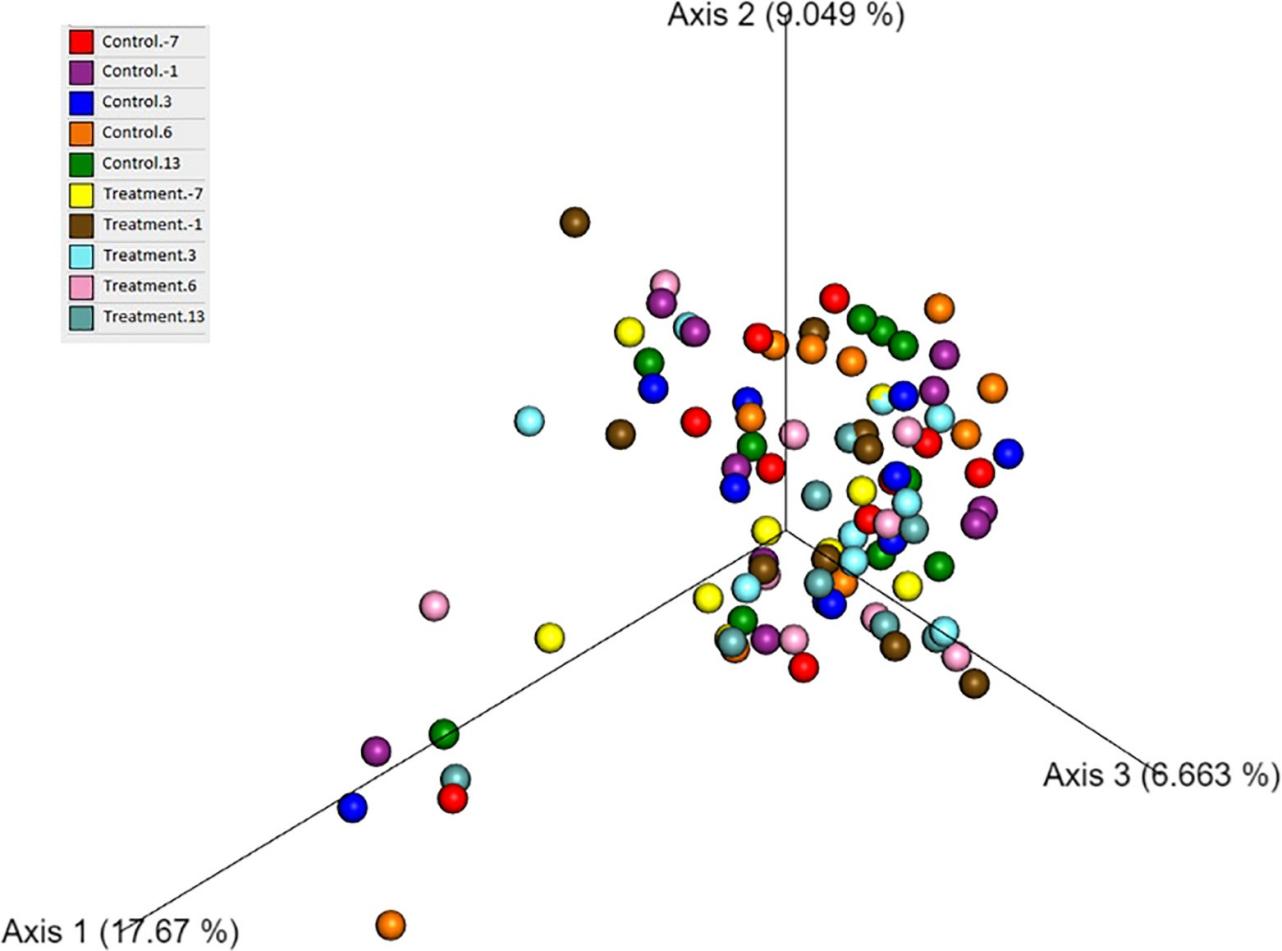
Principal coordinates analysis (PCoA) of microbial communities from fecal samples of dogs after administration of oral fenbendazole and from untreated controls. The figure shows a 3D PCoA plot based on unweighted UniFrac distances of 16S rRNA genes. The control and treatment group at each time point are represented by a different color. There is no clustering of dogs by treatment group or time point.

Beta diversity was assessed by PCoA plots and there was no significant difference in microbial communities across time points or between groups based on an ANOSIM (Fig 9).

### Univariate analysis

#### Part 1

Univariate statistics that reached statistical significance are listed in Table 2; however, no group was statistically different after correcting for multiple comparisons. The full data set is available in the supplementary data (S3 Table).

**Table 2:** Univariate analysis of 16S ribosomal RNA sequencing results of feces collected from six healthy adult research beagles with subclinical giardiasis and cryptosporidiosis. Dogs were administered a commercially available preparation of febantel combined with pyrantel and praziquantel (FPP) orally on days 0, 1 and 2 of the study. Different letters indicate statistically significant differences between time points (q < 0.05).

Bacterial group	Day -7		Day -3		Day 0		Day 4		Day 14		Day 21		Day -7 vs -3 vs 0 vs 4 vs 14 vs 21	
	Median	Range	Median	Range	Median	Range	Median	Range	Median	Range	Median	Range	P value	Q value
Phylum														
*Bacteroidetes*	1.2^a^	0.2–5.3	1^a^	0–5.6	3.9^a^	1.6–6.3	3.3^a^	0.8–5.3	2.4^a^	0.7–3.9	1.5^a^	0.3–7.6	0.0134	0.067
*Firmicutes*	26.7^a,b^	3.3–41.9	8.4^a,b^	1–38.2	0.7^a^	0–16	15.1^a,b^	8.9–27	13.3^a,b^	1.5–23.8	24.4^b^	4.7–35.6	0.0335	0.0837
Class														
*Coriobacteriia*	1.1^a,b^	0.2–2.5	0.3^a^	0–1.3	1.9^a,b^	0–5.7	2.3^b^	0.6–4.2	2.2^a,b^	0.7–3.8	1.4^a,b^	0.3–6.6	0.0311	0.1036
*Bacteroidia*	26.7^a,b^	3.3–41.9	8.4^a,b^	1–38.2	0.7^a^	0–16	15.1^a,b^	8.9–27	13.3^a,b^	1.5–23.8	24.4^b^	4.7–35.6	0.0134	0.067
*Bacilli*	17.1^a^	3.3–25.3	36.1^a,b^	10.1–65.6	41^b^	23.8–72.6	22.3^a,b^	12.9–45	33.7^a,b^	4–50.5	17.4^a,b^	2.1–61.3	0.0054	0.054
Order														
*Coriobacteriales*	1.1^a,b^	0.2–2.5	0.3^a^	0–1.3	1.9^a,b^	0–5.7	2.3^b^	0.6–4.2	2.2^a,b^	0.7–3.8	1.4^a,b^	0.3–6.6	0.0311	0.1244
*Bacteroidales*	26.7^a,b^	3.3–41.9	8.4^a,b^	1–38.2	0.7^a^	0–16	15.1^a,b^	8.9–27	13.3^a,b^	1.5–23.8	24.4^b^	4.7–35.6	0.0134	0.0804
*Lactobacillales*	14.6^a^	2.1–23.4	29.4^a,b^	8.6–65.6	37.9^b^	22.8–72.6	21.9^a,b^	12.9–44.6	33.3^a,b^	4–49.9	16^a,b^	1.9–61.3	0.008	0.0804
Family														
*Coriobacteriaceae*	1.1^a,b^	0.2–2.5	0.3^a^	0–1.3	1.9^a,b^	0–5.7	2.3^b^	0.6–4.2	2.2^a,b^	0.7–3.8	1.4^a,b^	0.3–6.6	0.0311	0.1555
*Bacteroidaceae*	15.7^a,b^	0.5–26.5	4.6^a,b^	0.4–23.3	0.4^a^	0–10.8	7.9^a,b^	3.3–14.4	7.6^a,b^	1.1–15.2	13.7^b^	0.7–21.4	0.0267	0.1555
*Prevotellaceae*	1.5^a^	0–4.5	2^a^	0–9.3	0^a^	0–0	2.9^a^	0.1–5.5	0.8^a^	0–6.5	1.5^a^	0–6.2	0.0464	0.1733
*Paraprevotellaceae*	8^a^	0.3–13.4	2.5^a,b^	0.1–4.7	0.4^b^	0–5	4.4^a,b^	0.6–9.2	2.8^a,b^	0–6.3	4.8^a,b^	0.6–10.9	0.009	0.1075
*Lactobacillaceae*	5^a^	1.5–18.2	21.5^b^	8–59.3	30.3^b^	12–62.2	9.1^a,b^	3.6–37.9	19.6^a,b^	1.9–39.3	8.1^a,b^	1.9–38.6	0.0041	0.1025
*Veillonellaceae*	3.9^b^	1.1–5.1	2.7^a,b^	0.2–4.2	0.3^a^	0–1	2.3^a,b^	2–5	2.7^a,b^	1–9.2	2.9^b^	2.4–5	0.0129	0.1075
Genus														
*Collinsella*	1.1^a,b^	0.2–2.2	0.3^a^	0–1.1	1.7^a,b^	0–5.6	2.3^b^	0.6–4.1	2.2^a,b^	0.7–3.7	1.4^a,b^	0.3–6.4	0.0311	0.1822
*Bacteroides*	15.7^a,b^	0.5–26.5	4.6^a,b^	0.4–23.3	0.4^a^	0–10.8	7.9^a,b^	3.3–14.4	7.6^a,b^	1.1–15.2	13.7^b^	0.7–21.4	0.0267	0.1822
*Prevotella*	1.5^a^	0–4.5	2^a^	0–9.3	0^a^	0–0	2.9^a^	0.1–5.5	0.8^a^	0–6.5	1.5^a^	0–6.2	0.0464	0.1927
*Prevotella*	3.4^a^	0.3–8	0.3^a^	0–2.9	0.2^a^	0–2.5	3^a^	0.2–7.5	2^a^	0–5.3	2.9^a^	0.6–7.1	0.005	0.1025
*Lactobacillus*	5^a^	1.5–18.2	21.5^b^	8–59.3	30.3^b^	12–62.2	9.1^a,b^	3.6–37.9	19.6^a,b^	1.9–39.3	8.1^a,b^	1.9–38.6	0.0041	0.1025
*Blautia*	3.2^a^	0.5–6.7	3.7^a^	1.2–5.7	6.5^a^	0–12	7.9^a^	2.2–9.1	5.5^a^	2.4–9.1	4.6^a^	1.9–10.1	0.0375	0.1922
*Megamonas*	2.9^a^	0.5–4.5	2.1^a,b^	0–2.9	0.2^b^	0–1	1.4^a,b^	0.6–4.7	1.4^a,b^	0.4–9.2	1.3^a,b^	0.4–4.6	0.0156	0.1664
*Phascolarctobacterium*	0.5^a,b^	0–2.2	0.1^a,b^	0–2.4	0.1^a^	0–0.1	0.7^a,b^	0–1.2	0.7^a,b^	0–1.5	1.4^b^	0–2.5	0.0178	0.1664
Unclassified *Erysipelotrichaceae*	0.3^a^	0–0.9	0.2^a^	0–0.6	1.2^a^	0–3.2	1.8^a^	0.5–3.7	0.7^a^	0–3.2	0.7^a^	0.2–1.7	0.047	0.1927
Unclassified *Succinivibrionaceae*	4.2^a^	1.4–7.9	1.2^a,b^	0.6–5.8	0.1^b^	0–4.4	1^a,b^	0–3	0.8^a,b^	0–1.4	2^a,b^	0–3.3	0.0203	0.1664
Species														
*Collinsella stercoris*	1.1^a,b^	0.2–2.1	0.3^a^	0–1.1	1.7^a,b^	0–5.6	2.3^b^	0.6–4.1	2.2^a,b^	0.7–3.7	1.4^a,b^	0.3–6.2	0.0311	0.2043
Unclassified *Bacteroides*	6.5^a,b^	0.1–12	1.6^a,b^	0–8.7	0.3^a^	0–4.9	4.3^a,b^	0.7–8.4	2.5^a,b^	0–7.6	7.1^b^	0.3–9.9	0.0088	0.1349
*Prevotella copri*	1.5^a^	0–4.5	2^a^	0–9.3	0^a^	0–0	2.9^a^	0.1–5.5	0.8^a^	0–6.5	1.5^a^	0–6.2	0.0464	0.2128
Unclassified *Prevotella*	3.4^a^	0.3–8	0.3^a^	0–2.9	0.2^a^	0–2.5	3^a^	0.2–7.5	2^a^	0–5.3	2.9^a^	0.6–7.1	0.005	0.115
Unclassified *Lactobacillus*	5^a^	1.5–18.2	21.5^b^	8–59.3	30.3^b^	12–62.2	9.1^a,b^	3.6–37.9	19.6^a,b^	1.9–39.3	8.1^a,b^	1.9–38.6	0.0041	0.115
*Ruminococcus gnavus*	1.3^a,b^	0.4–4.6	1.5^a,b^	0.6–3.3	0.9^a^	0–1.5	1.7^a,b^	0.5–4	2.7^b^	1.2–5	1.7^a,b^	0–3.2	0.0377	0.2128
Unclassified *Megamonas*	2.9^a^	0.5–4.5	2.1^a,b^	0–2.9	0.2^b^	0–1	1.4^a,b^	0.6–4.7	1.4^a,b^	0.4–9.2	1.3^a,b^	0.4–4.6	0.0156	0.1556
Unclassified *Phascolarctobacterium*	0.5^a,b^	0–2.2	0.1^a,b^	0–2.4	0.1^a^	0–0.1	0.7^a,b^	0–1.2	0.7^a,b^	0–1.5	1.4^b^	0–2.5	0.0178	0.1556
Unclassified *Erysipelotrichaceae*	0.3^a^	0–0.9	0.2^a^	0–0.6	1.2^a^	0–3.2	1.8^a^	0.5–3.7	0.7^a^	0–3.2	0.7^a^	0.2–1.7	0.047	0.2128
Unclassified *Succinivibrionaceae*	4.2^a^	1.4–7.9	1.2^a,b^	0.6–5.8	0.1^b^	0–4.4	1^a,b^	0–3	0.8^a,b^	0–1.4	2^a,b^	0–3.3	0.0203	0.1556

#### Part 2

Univariate statistics showed that the family *Clostridiaceae* was significantly different (*p =* 0.032) over time in the control group. However, this difference failed to reach significance after correcting for multiple comparisons (*q* = 0.608). Table 3 shows the groups that had significant change in abundance over time in the dogs treated with fenbendazole. No group changed significantly over time after correcting for multiple comparisons. Available in the supplementary data are the controls over time (S4 Table) and treatment with fenbendazole over time (S5 Table).

**Table 3:** Univariate analysis of 16S ribosomal RNA sequencing results of feces collected from 9 healthy staff-owned dogs treated with fenbendazole. Fenbendazole (50 mg/kg) was administered to dogs in the treatment group on days 0–4. Different letters indicate statistically significant differences between time points (q < 0.05).

Bacterial group	Day -7		Day -1		Day 3		Day 6		Day 13		Day -7 vs -1 vs 3 vs 6 vs 13	
	Median	Range	Median	Range	Median	Range	Median	Range	Median	Range	P value	Q value
Family												
*Lachnospiraceae*	6^a^	3.8–9	8.1^a^	2.6–12.6	11.6^a^	4.4–20.4	8.3^a^	4.1–16.1	8.3^a^	5.3–16.3	0.0368	0.72
Genus												
*Clostridium*	0^a,b^	0–0.7	0^a^	0–0.2	0^a,b^	0–2	0^a,b^	0–4	0.6^b^	0–3.5	0.0236	0.3245
*Blautia*	1.9^a^	0.9–3.5	2.1^a,b^	0.8–6.5	4.5^b^	1.2–6.4	2.5^a,b^	0.7–6.3	2.6^a,b^	0.3–5	0.0295	0.3245
*Oscillospira*	0^a^	0–0.4	0^a^	0–0.3	0.1^a^	0–1	0^a^	0–0.5	0^a^	0–0.7	0.0457	0.377
*Helicobacter*	0.6^a,b^	0–3.5	1.1^a^	0–3.3	0.6^a,b^	0–4.1	0.7^a,b^	0–4	4.2^b^	0.1–7	0.0209	0.3245
Species												
Unclassified *Blautia*	0.3^a^	0–0.8	0.5^a^	0–3.2	1.2^a^	0–3.6	1^a^	0–3.2	0.5^a^	0–1.7	0.0224	0.5082

## Discussion

Our study only found minimal changes in the fecal microbiome in dogs before and after administration of either of the two anthelmintics (i.e., combination product febantel, pyrantel, and praziquantel or fenbendazole). To the authors’ knowledge this is the first study to evaluate the effect of either anthelmintic treatment on the fecal microbiome of dogs. A recent pilot study identified a subtle effect on the gastrointestinal microbiome after administration of a single dose of moxidectin/praziquantel in horses [30]. Another study found alterations in the gastrointestinal microbiota in Amur tigers after they received tablets containing fenbendazole and ivermectin [31]. However, most studies in other species did not find major effects of various anthelmintic agents on the intestinal microbiome. Specifically, fenbendazole did not have an effect on the hindgut microbiota of horses [20] or the gut microbiome of mice [21] and a moxidectin praziquantel combination product did not have a major effect on the fecal microbiota of horses [22]. Differences between the results of these studies may be attributable to species differences, different active ingredients studied or dosages and the different definitions of what constitutes an important change in the gastrointestinal microbiome.

Quantitative PCR results showed that the fecal abundance of *Fusobacterium* increased between days 0 and 4 in research beagles with subclinical giardiasis and cryptosporidiosis. However, this change was transient, the abundance of the other bacterial groups evaluated did not change and therefore in the authors’ opinion, this finding is unlikely to be of clinical importance. 16S ribosomal RNA sequencing showed that species richness and microbial diversity did not significantly change after FPP administration. After correcting for multiple comparisons there were no changes in individual bacterial phyla, classes, orders, families, genera, or species after treatment with FPP. This is in contrast to the major and more long-standing changes in the fecal microbiome of healthy dogs administered metronidazole or tylosin, a macrolide antibiotic [14, 32]. Taken together these results only show minimal changes in the fecal microbiota of research beagles with subclinical giardiasis and cryptosporidiosis infection when they were administered oral FPP at a standard dose for three days. However, the authors cannot rule out such an effect if the FPP was given at higher doses or for a longer period of time. Our results concur with those reported in other species, where fenbendazole did not have an effect on the hindgut microbiota of horses [20] or the gut microbiome of mice [21] and a moxidectin praziquantel combination product did not have a major effect on the fecal microbiota of horses [22].

In healthy staff-owned dogs administered fenbendazole, there was a significant effect of treatment group on the fecal abundance of *Fusobacterium* and *E*. *coli*. However, there was no significant interaction between time point and treatment suggesting any difference was due to baseline differences between groups rather than as an effect of fenbendazole administration. Furthermore, after correcting for multiple comparisons, there were no significant difference in the fecal abundance of either bacterial group between treated and control dogs at any of the time points. Again, microbial diversity was not altered by fenbendazole administration, which is in contrast to metronidazole which significantly altered microbial structure and diversity [14]. After correcting for multiple comparisons there were no changes in individual bacterial phyla, classes, orders, families, genera, or species over time in the group treated with fenbendazole or the control group. Again, this is in contrast to the major and more long-standing changes in the fecal microbiome of healthy dogs administered metronidazole or the macrolide antibiotic, tylosin [14, 32]. Taken together these results only show minimal changes in the fecal microbiota of healthy staff owned dogs when they were administered oral fenbendazole at a standard dose for five days. However, the authors cannot rule out such an effect if the fenbendazole was given at higher doses or for a longer period of time. Our results concur with those reported in other species, where fenbendazole did not have an effect on the hindgut microbiota of horses [20] or the gut microbiome of mice [21].

Antibiotics have a profound impact on the microbiota and in humans and use during childhood can lead to abnormal development of the intestinal microbiome [9]. Because most dogs clinically affected by *Giardia* spp. are young, it seems prudent that treatment should be targeted towards treating the disease without negatively impacting the patient’s developing microbiota. Based on the results of this study, treatment for clinical giardiasis with FPP or fenbendazole may be less likely to negatively impact the fecal microbiome in dogs than metronidazole.

There were only minimal changes in the fecal microbiome of dogs infected with *Giardia* spp. and *C*. *canis* before and after treatment with FPP. Additionally, the results of the qPCR assays were similar to those previously found in healthy dogs [25]. This finding conflicts a previous report in which 17 non-diarrheic dogs with *Giardia* spp. infection were found to have a significantly different bacterial genera present in the microbiome compared to the 23 dogs not infected with *Giardia* spp. [6]. The lack of difference in our study could be due to the relatively small sample size leading to a type II error or use of dogs from a research colony. Furthermore, we cannot make direct comparisons between the sequencing of the research beagles and the healthy staff-owned dogs as the sequencing was performed as separate runs.

One dog administered FPP was positive for *Giardia* cysts and *C*. *canis* oocysts prior to treatment, negative on post-treatment days 4 and 7, and positive on day 14. It cannot be determined from the data whether these were persistent infections or were re-infections. In addition, these research beagles and most dogs are infected with *C*. *canis* which is not considered a primary pathogen in people [10].

This is the first study to show that neither FPP nor fenbendazole cause substantial changes in the fecal microbiome of dogs. However, it is subject to several limitations. Firstly, there was a lack of standardization of drugs used between the two parts of the study. FPP was used in part 1 because febantel when combined with pyrantel has a synergistic effect in treatment for *Giardia* spp. compared to febantel alone [33]. Fenbendazole was chosen as the study drug in part 2 because it is commonly used by the authors as an empiric anthelmintic in dogs with chronic enteropathies and is an effective treatment for giardiasis [34]. No placebo was administered to the control dogs in part 2 of the study; however, this is unlikely to have significantly affected the results since aside from fecal scores no subjective outcome measures were used. Additionally, diet was not standardized in part 2 of the study; however, there was no significant difference in the baseline results between the treatment and control groups so this is unlikely to have had a major effect on the results. Furthermore, in a previous study, there were no changes in the fecal microbiome in dogs that were transitioned to a commercial hydrolyzed diet [14]. Finally, there was no control group in part 1 of the study because all dogs were co-infected with *Giardia* spp. and *C*. *canis*. Therefore, it was elected to treat all dogs with FPP.

## Conclusions

FPP administration was associated with minimal alterations of the fecal microbiome of research beagles with subclinical giardiasis and cryptosporidiosis. Fenbendazole administration was associated with minimal alterations of the fecal microbiome of healthy staff owned dogs.

## Supporting information

S1 Questionnaire Owner questionnaire completed for client owned dogs that received either either fenbendazole or no treatment. This questionnaire was completed prior to enrollment to assess each dog’s health status and establish fulfillment of inclusion criteria.
(DOCX)

S1 Table Fecal scores and parasitology results of research beagles with subclinical giardiasis and cryptosporidiosis administered a commercially available preparation of febantel combined with pyrantel and praziquantel (FPP) orally on days 0, 1 and 2 of the study. All fecal scores were <4 with the exception of one dog on day 0 that had a fecal score of 4, one dog on all post-treatment days, and one dog on day 14. When all pretreatment results are combined, it was shown that all 6 dogs were co-infected with *C*. *canis*.
(DOCX)

S2 Table Fecal dysbiosis index and fecal abundance of bacterial groups in healthy dogs after administration of oral fenbendazole and in healthy controls. A mixed model was used to assess the effect of treatment group (fenbendazole vs. control), time point (days -7 and -1 are pre-treatment, day 3 during the treatment with fenbendazole (FBZ), and days 6 and 13 are post-treatment), and their effect on the fecal dysbiosis index and the fecal abundance of bacterial groups measured by quantitative PCR. *for these groups there was a significant effect of treatment group but upon post hoc testing (after correcting for multiple comparisons) there was no significant difference between groups at any of the time points.
(DOCX)

S3 Table Univariate analysis of 16S ribosomal RNA sequencing results of feces collected from six healthy adult research beagles with subclinical giardiasis and cryptosporidiosis. Dogs were administered a commercially available preparation of febantel combined with pyrantel and praziquantel (FPP) orally on days 0, 1 and 2 of the study.
(XLSX)

S4 Table Univariate analysis of 16S ribosomal RNA sequencing results of feces collected from 10 healthy staff-owned dogs. (XLSX)

S5 Table Univariate analysis of 16S ribosomal RNA sequencing results of feces collected from 9 healthy staff-owned dogs treated with fenbendazole. Fenbendazole (50 mg/kg) was administered to dogs in the treatment group on days 0–4.
(XLSX)
