# Supplementary material for: Evaluation of the effects of anthelmintic administration on the fecal microbiome of healthy dogs with and without subclinical Giardia spp. and Cryptosporidium canis infections
Source: PLoS One. 2020 Feb 6;15(2):e0228145. doi: 10.1371/journal.pone.0228145 (PMC7004322; doi:10.1371/journal.pone.0228145)
Supplement: S1 Questionnaire — This questionnaire was completed prior to enrollment to assess each dog’s health status and establish fulfillment of inclusion criteria. (DOCX) [file pone.0228145.s001.docx]

Client Questionnaire (circle yes or no when appropriate)

1. Has your dog had any episodes of gastrointestinal upset (ie- vomiting, diarrhea, or anorexia) that lasted longer than 2 consecutive days within the last 3 months?

Yes No

If yes, please explain:________________________________________

1. Has your dog received antibiotics in the past 6 months?

Yes No

If so, what antibiotic and what was the indication?________________

_________________________________________________________

1. Does your dog have any history of systemic illness?

Yes No

If yes, please explain:________________________________________

1. Is your dog receiving any medications other than routine heartworm, flea, tick prevention?

Yes No

If yes, please explain:________________________________________

1. Please list current preventative medications your dog takes and date of last administration:

Heartworm, Brand:_______________ Date: _______________

Tick, Brand:_______________ Date: _______________

Flea, Brand:_______________ Date: _______________
