# Supplementary material for: Evaluation of the effects of anthelmintic administration on the fecal microbiome of healthy dogs with and without subclinical Giardia spp. and Cryptosporidium canis infections
Source: PLoS One. 2020 Feb 6;15(2):e0228145. doi: 10.1371/journal.pone.0228145 (PMC7004322; doi:10.1371/journal.pone.0228145)
Supplement: S1 Table — All fecal scores were <4 with the exception of one dog on day 0 that had a fecal score of 4, one dog on all post-treatment days, and one dog on day 14. When all pretreatment results are combined, it was shown that all 6 dogs were co-infected with C. canis. (DOCX) [file pone.0228145.s002.docx]

| **Dog ID** | **Day** | **Fecal Score** | **Fecal IFA** | **Fecal Flotation** |
| --- | --- | --- | --- | --- |
| A | -7 | 3 | *Giardia* | - |
| B | -7 | 3 | *Giardia* and *Cryptosporidium* | - |
| C | -7 | 2 | *Giardia* and *Cryptosporidium* | - |
| D | -7 | 3 | *Giardia* | - |
| E | -7 | 3 | *Giardia* and *Cryptosporidium* | - |
| F | -7 | 2 | *Giardia* | - |
| A | -3 | 3 | Negative | Negative |
| B | -3 | 3 | *Giardia* | Negative |
| C | -3 | 3 | Negative | Negative |
| D | -3 | 2 | *Cryptosporidium* | Negative |
| E | -3 | 2 | Negative | Negative |
| F | -3 | 3 | *Giardia* and *Cryptosporidium* | Negative |
| A | 0 | 2 | *Giardia* and *Cryptosporidium* | *Giardia* alone |
| B | 0 | 2 | *Giardia* | Negative |
| C | 0 | 2 | Negative | Negative |
| D | 0 | 3 | Negative | Negative |
| E | 0 | 2 | *Cryptosporidium* | Negative |
| F | 0 | 4 | *Giardia* | Negative |
| A | 4 | 3 | Negative | Negative |
| B | 4 | 2 | Negative | Negative |
| C | 4 | 2 | Negative | Negative |
| D | 4 | 4 | Negative | Negative |
| E | 4 | 2 | Negative | Negative |
| F | 4 | 3 | Negative | Negative |
| A | 7 | 2 | Negative | Negative |
| B | 7 | 3 | Negative | Negative |
| C | 7 | 3 | Negative | Negative |
| D | 7 | 4 | Negative | Negative |
| E | 7 | 3 | Negative | Negative |
| F | 7 | 3 | Negative | Negative |
| A | 14 | 2 | *Giardia* and *Cryptosporidium* | Negative |
| B | 14 | 2 | Negative | Negative |
| C | 14 | 4 | Negative | Negative |
| D | 14 | 4 | Negative | Negative |
| E | 14 | 3 | Negative | Negative |
| F | 14 | 3 | Negative | Negative |
| A | 21 | 2 | Negative | Negative |
| B | 21 | 3 | Negative | Negative |
| C | 21 | 3 | Negative | Negative |
| D | 21 | 4 | Negative | Negative |
| E | 21 | 2 | Negative | Negative |
| F | 21 | 3 | Negative | Negative |
