# Supplementary material for: Evaluation of the effects of anthelmintic administration on the fecal microbiome of healthy dogs with and without subclinical Giardia spp. and Cryptosporidium canis infections
Source: PLoS One. 2020 Feb 6;15(2):e0228145. doi: 10.1371/journal.pone.0228145 (PMC7004322; doi:10.1371/journal.pone.0228145)
Supplement: S2 Table — A mixed model was used to assess the effect of treatment group (fenbendazole vs. control), time point (days -7 and -1 are pre-treatment, day 3 during the treatment with fenbendazole (FBZ), and days 6 and 13 are post-treatment), and their effect on the fecal dysbiosis index and the fecal abundance of bacterial groups measured by quantitative PCR. *for these groups there was a significant effect of treatment group but upon post hoc testing (after correcting for multiple comparisons) there was no significant difference between groups at any of the time points. (DOCX) [file pone.0228145.s003.docx]

| Bacterial group | Effect of treatment group (*p*-value) | Effect of time point (*p*-value) | Effect of interaction between treatment group and time point (*p*-value) |
| --- | --- | --- | --- |
| Dysbiosis index | 0.4950 | 0.0957 | 0.8578 |
| Universal bacterial | 0.4775 | 0.2541 | 0.4384 |
| *Faecalibacterium* | 0.7253 | 0.1339 | 0.9199 |
| *Turicibacter* | 0.8256 | 0.2927 | 0.8643 |
| *Streptococcus* | 0.8612 | 0.3947 | 0.9413 |
| *Escherichia coli* | 0.0363^*^ | 0.0585 | 0.8236 |
| *Blautia* | 0.9268 | 0.2642 | 0.8706 |
| *Fusobacterium* | 0.0401^*^ | 0.7927 | 0.3624 |
| *Clostridium hiranonis* | 0.3210 | 0.9179 | 0.2454 |
| *Clostridioides difficile* | 0.2480 | 0.8153 | 0.8153 |
| *Clostridium perfringens* | 0.7289 | 0.5363 | 0.6500 |
